# Evaluating signals of oil spill impacts, climate, and species interactions in Pacific herring and Pacific salmon populations in Prince William Sound and Copper River, Alaska

**DOI:** 10.1371/journal.pone.0172898

**Published:** 2017-03-15

**Authors:** Eric J. Ward, Milo Adkison, Jessica Couture, Sherri C. Dressel, Michael A. Litzow, Steve Moffitt, Tammy Hoem Neher, John Trochta, Rich Brenner

**Affiliations:** 1 Conservation Biology Division, Northwest Fisheries Science Center, National Marine Fisheries Service, National Oceanic and Atmospheric Administration, Seattle Washington, United States of America; 2 School of Fisheries and Ocean Sciences, University of Alaska Fairbanks, Juneau, Alaska, United States of America; 3 National Center for Ecological Analysis and Synthesis, Santa Barbara, California, United States of America; 4 Alaska Department of Fish and Game, Division of Commercial Fisheries, Juneau, Alaska, United States of America; 5 Farallon Institute for Advanced Ecosystem Research, Petaluma, California, United States of America; 6 Alaska Department of Fish and Game, Division of Commercial Fisheries, Cordova, Alaska, United States of America; 7 Kasitsna Bay Laboratory, National Ocean Service, National Oceanic and Atmospheric Administration, Homer, Alaska, United States of America; 8 School of Aquatic and Fishery Sciences, University of Washington, Seattle, Washington, United States of America; Universidade de Aveiro, PORTUGAL

## Abstract

The *Exxon Valdez* oil spill occurred in March 1989 in Prince William Sound, Alaska, and was one of the worst environmental disasters on record in the United States. Despite long-term data collection over the nearly three decades since the spill, tremendous uncertainty remains as to how significantly the spill affected fishery resources. Pacific herring (*Clupea pallasii*) and some wild Pacific salmon populations (*Oncorhynchus spp*.) in Prince William Sound declined in the early 1990s, and have not returned to the population sizes observed in the 1980s. Discerning if, or how much of, this decline resulted from the oil spill has been difficult because a number of other physical and ecological drivers are confounded temporally with the spill; some of these drivers include environmental variability or changing climate regimes, increased production of hatchery salmon in the region, and increases in populations of potential predators. Using data pre- and post-spill, we applied time-series methods to evaluate support for whether and how herring and salmon productivity has been affected by each of five drivers: (1) density dependence, (2) the EVOS event, (3) changing environmental conditions, (4) interspecific competition on juvenile fish, and (5) predation and competition from adult fish or, in the case of herring, humpback whales. Our results showed support for intraspecific density-dependent effects in herring, sockeye, and Chinook salmon, with little overall support for an oil spill effect. Of the salmon species, the largest driver was the negative impact of adult pink salmon returns on sockeye salmon productivity. Herring productivity was most strongly affected by changing environmental conditions; specifically, freshwater discharge into the Gulf of Alaska was linked to a series of recruitment failures—before, during, and after EVOS. These results highlight the need to better understand long terms impacts of pink salmon on food webs, as well as the interactions between nearshore species and freshwater inputs, particularly as they relate to climate change and increasing water temperatures.

## Introduction

Impacts of human-caused environmental disasters—such as oil spills or nuclear accidents—are often realized immediately, but may also result in lasting change over decades or longer [1,2]. Detecting impacts of these disasters relies on dedicated funding and long-term monitoring; however, attributing change to these singular catastrophic events may be difficult when environmental and ecological variables measured in long-term monitoring efforts are simultaneously affected by other external pressures (e.g., climate variability, removals from fishing). Inference about impacts may be further complicated by how species are prioritized for monitoring, and how the allocation of monitoring effort is distributed in space and time [3].

**Fig 1:**
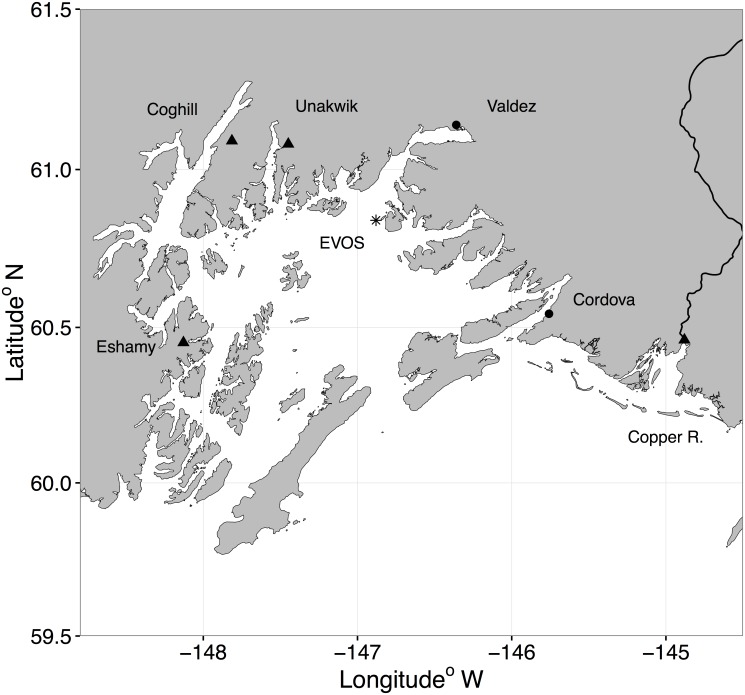
Map of Prince William Sound, and the adjacent Copper River Alaska. Triangles indicate the location of wild salmon stocks included in our analyses, circles show towns, and the asterisk shows where the *Exxon Valdez* ran aground in 1989.

One of the most well-known and documented environmental catastrophe with available long-term monitoring studies is the *Exxon Valdez* oil spill (EVOS). On March 23, 1989, the oil tanker *Exxon Valdez* ran aground in Prince William Sound (PWS), in southcentral Alaska (Fig 1). This region represents an ecosystem where multiple complex interactions between environmental conditions and terrestrial, nearshore, and pelagic components drive high rates of productivity [4,5]. The tanker spilled an estimated 42 million liters of crude oil into the area, contaminating marine waters for more than 800 km to the southwest [6–8,8–10]. Nearly 40 percent of the oil landed on beaches within PWS, affecting over 780 km of shoreline [11]. In the more than 25 years since the EVOS disaster, resource managers and researchers from federal, state, university, and non-profit organizations have collected a vast amount of information to quantify the effects of the spill and evaluate recovery of injured resources. Despite these monitoring efforts, the direct and indirect environmental impacts attributable to EVOS are still hotly debated by the scientific community [12,13].

The most scrutinized effects of EVOS have been related to direct exposure effects of oil, affecting species or populations closely associated in space and time with the obvious presence of oil. Clean-up efforts, combined with the dynamic marine tidal and weather patterns, were expected to remove or displace much of the spilled oil from the environment in several years [14]. Studies conducted a decade after EVOS estimated the remaining oil to be < 1% of that originally estimated, but lingering toxicity effects were still considered to be a concern [15]. More recent work has provided a mechanism by which this residual oil can have chronic effects on species that depend upon nearshore rearing and spawning areas. In particular, species such as Pacific herring (*Clupea pallasii*) and pink salmon (*Oncorhynchus gorbuscha*) that use nearshore habitats may be affected by crude oil through physiological defects that lead to reduced growth rates and higher larval and juvenile mortality [16]. While experimental studies have found support for toxic effects of oil on individuals, a larger challenge is identifying persistent effects at the population level, where duration and magnitude of oil exposure is unknown.

Herring and multiple species of salmon have been the focus of a large number of research studies in PWS, both because of their value to commercial fisheries and because of population-level changes observed in PWS during or after the EVOS disaster. For example, the PWS population of herring suffered a well-documented collapse in 1993, resulting in a closure of the commercial fishery, and to date, the population has not recovered [17,18]. Similarly, low returns of pink salmon to PWS also occurred in 1992 and 1993, [19,20], and Willette et al. [21] proposed that Coghill Lake sockeye had been impacted by EVOS as juveniles in the nearshore environment. While the majority of studies investigating EVOS impacts have not found strong effects [12,22], a number of confounding hypotheses have been proposed for explaining observed changes in fish population dynamics; these include disease, variation in the ocean environment, changes in spawning habitat, changes in interactions between species, intraspecific density dependence, and increases in predation from higher trophic level species, such as marine birds and mammals [12,13,23].

### Alternative hypotheses for herring and salmon declines

Over the last four decades, the PWS region has experienced a number of changes or regime shifts that may have also affected the productivity of species such as herring and salmon. In 1976–77 the coastal North Pacific experienced a dramatic increase in temperature that coincided with the large-scale realignment of marine communities [24,25]. Like the rest of the North Pacific Ocean, water temperatures have also been gradually increasing, resulting in anomalously high values [26,27]. Of particular interest to this study was the climate regime shift that occurred in 1989, which led to an ecosystem state thought to be less productive [28], thereby confounding assessments of the direct impact of the oil spill. Periods of warm and cool regimes in ocean temperature have also been correlated with changes in freshwater input, wind patterns, and water column stability that lead to shifts in marine productivity [29–32]. Over the past 40 years, the northern Gulf of Alaska has undergone a general warming and freshening in the upper 100 m of the water column; with an increase in salinity in depths between 100–200 m. This suggests that vertical stratification in the upper water column in the Gulf of Alaska has increased substantially [33]. In coincidence with the changes in the physical environment, higher water temperatures impact metabolism and consequently growth, energy demands, and ultimately, behavior and survival of larval and juvenile fishes [31,34]. Thus, these environmental changes in bottom-up forcing resulting from changes in temperature and productivity add to the variability in survival of both adult and juvenile herring and salmon.

In response to poor runs of wild salmon during the late 1960s and early 1970s, state and non-profit hatcheries began releasing salmon into areas of PWS in 1976 [35,36], with possible consequences to wild salmon and herring. A substantial increase in hatchery pink salmon production occurred during the late 1980s, just prior to the spill; thus representing another potential confounding effect (S1 Fig). Ecological impacts of this change have been speculated to impact both wild salmon and forage fish that compete for similar prey resources or serve as prey to adult returning fish [22,34,37,38]. Studies from other regions in the Northeast Pacific have demonstrated evidence for dietary overlap between pink salmon and herring [39] and pink salmon in particular are known to consume a diversity of prey items in the marine environment, from zooplankton to herring and other fish [40,41], and compete with salmon species including chum (*O*. *keta*), Chinook (*O*. *tshawytscha*) and sockeye salmon (*O*. *nerka*) [42].

In addition to the possibility of increased competition or predation from hatchery released salmon, the population dynamics of herring and salmon in PWS may also have been affected by other predators. Potential predators include populations of humpback whales (*Megaptera novaeangliae*) or piscivorous marine birds [23,43,44]. Effects of these predators on herring and salmon may be direct, or indirect through apparent competition. Combined with climate drivers, recoveries of these predators throughout the Northeast Pacific Ocean have the ability to alter the ecosystem state relative to the 1980s (e.g. alternating from a period of high productivity and low predation to low productivity and high predation).

### Linking covariates to herring and salmon productivity

Previous studies on herring and salmon juvenile mortality in PWS have focused on finding effects within a narrow geographic or temporal window [45–47] less on impacts at the population or stock level. Additionally, previous testing and review of hypotheses on the collapse and recovery failure of PWS herring primarily focus on adult survival [3,22,48]. Because of relatively high uncertainty concerning what factors are primarily responsible for variation in herring and salmon recruitment, we adopted a statistical approach to evaluate multiple hypotheses about lasting effects of EVOS, and long term productivity change in PWS and the adjacent Copper River. The purpose of our analysis is to synthesize and review the working hypotheses about changes in productivity, and to use time series methods to evaluate the data support for each, 25 years after the oil spill. These hypotheses include: (1) effects of intraspecific density dependence, or increasing per capita population growth rate at decreasing population density (2) immediate and/or prolonged impacts of the EVOS event, (3) impacts of changing environmental conditions, (4) effects of interspecific competition on juvenile fish, and (5) effects of competition and predation from adult fish or, in the case of herring, humpback whales.

## Methods

### Data

**Fig 2:**
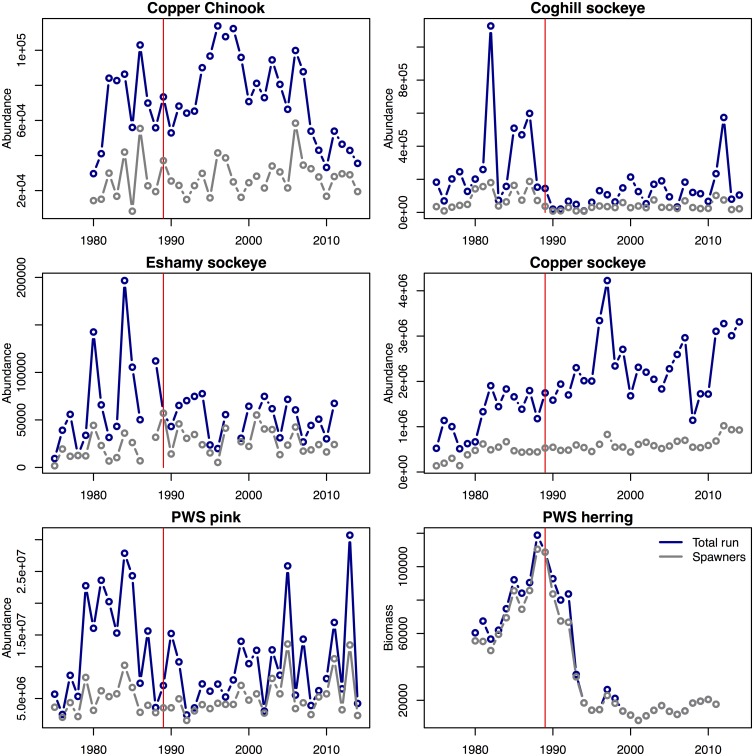
Time series of total run and escapement (or spawning biomass, herring). Total population size and escapement (salmon, in numbers of fish) or total population biomass and spawning stock biomass (spawning herring, in metric tons) for the six populations and four species in our analysis. Harvest for each population can be interpreted as the difference between total (black) and spawning (grey) lines. Red vertical lines are used to indicate 1989 (corresponding to the year of the EVOS event).

**Fig 3:**
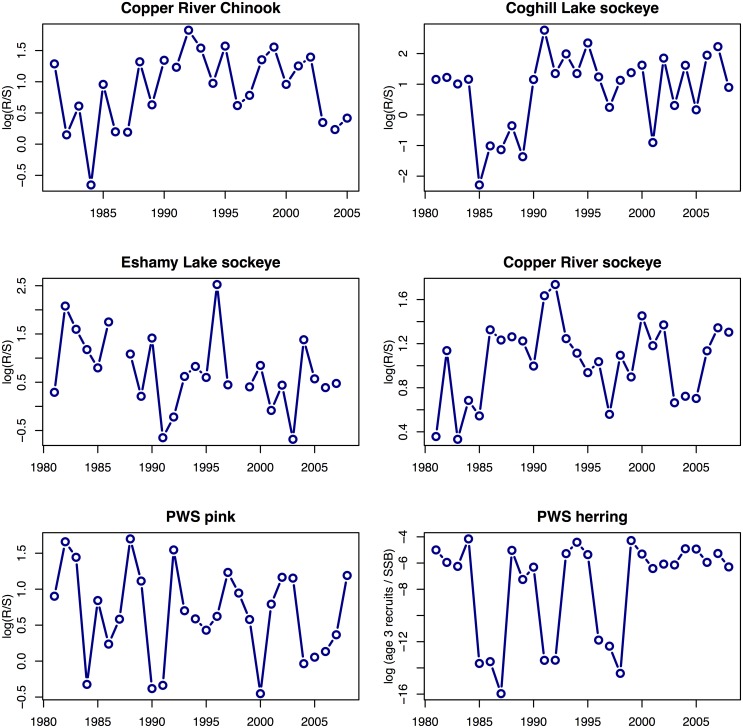
Time series of recruits-per-spawner relationship for data included in our analysis. Raw data are shown for the years included in our analysis. R = recruits, S = spawners, SSB = spawning stock biomass, age-3 recruits = millions of mature and immature age-3 herring, and PWS = Prince William Sound.

We examined the evidence of drivers affecting recruitment in Pacific herring and three species of salmon within the Prince William Sound management area: Chinook salmon, pink salmon, and sockeye salmon (Fig 2). Specifically, we examined the amount of recruitment divided by the total reproductive component of the population, measured as spawning biomass for herring or as the number of spawning adults for salmon (Fig 3); this ratio of recruits to the spawning population is referred to as productivity. We conducted the analysis for each species separately, using the longest time series possible that also allowed similar drivers to be compared. For Pacific herring, we analyzed recruits per spawning stock biomass (R/SSB) from PWS as the response, where recruits (defined as the number of mature and immature age-3 fish) and SSB are estimated from the Alaska Department of Fish and Game (ADF&G) age structured stock assessment model (ADF&G, pers. comm., https://github.com/NCEAS/pfx-covariation-pws) for brood years 1981–2011. For each of three salmon species, we calculated the total adult returns, summed across all ages of return, which were the offspring of spawning adults in a particular year (i.e., total brood year returns per spawner). For Chinook salmon, we used wild spawning escapements and wild brood year returns from the Copper River for brood years 1981–2005. For wild pink salmon, we used estimates of total run size and escapement in PWS. Due to the harvest of migrating fish, productivity of PWS pink salmon can only be calculated for the entire area and not for individual stocks or districts. Finally, for wild sockeye salmon, we examined spawner and recruitment data from three populations (Coghill Lake and Eshamy Lake in PWS and the adjacent Copper River), both separately and combined. These salmon stocks were included based on the availability of data on recruitment and age structure and because they transit PWS—or have the possibility to transit PWS—as juveniles and/or as returning adults. Data from ADF&G and others suggest that adult and juvenile salmon from throughout PWS use the southwestern passages of PWS as a primary migratory corridor [49–51], which were heavily oiled during EVOS [6,7]. The adjacent Copper River was not directly oiled during EVOS; however, we included Copper River stocks in our analyses because of the potential for juvenile salmon from the Copper River to be pushed into PWS by the Alaska Coastal Current [52] and into oiled areas by the cyclonic current within PWS [53]. It is not known if adult salmon returning to the Copper River transit through PWS. Limited data are also included for other populations in the region (PWS wild chum salmon, Unakwik district sockeye salmon, S4 and S5 Figs, https://github.com/NCEAS/pfx-covariation-pws) but missing age and escapement data prevents estimation of recruitment. All salmon data are provided in ADF&G reports [54,55].

For each of the five hypothesized mechanisms included in our analyses, we were interested in quantifying the data support for each hypothesis and species. The five hypotheses are explained in detail as follows:

#### Hypothesis 1: Patterns in productivity are driven by density dependence

**Fig 4:**
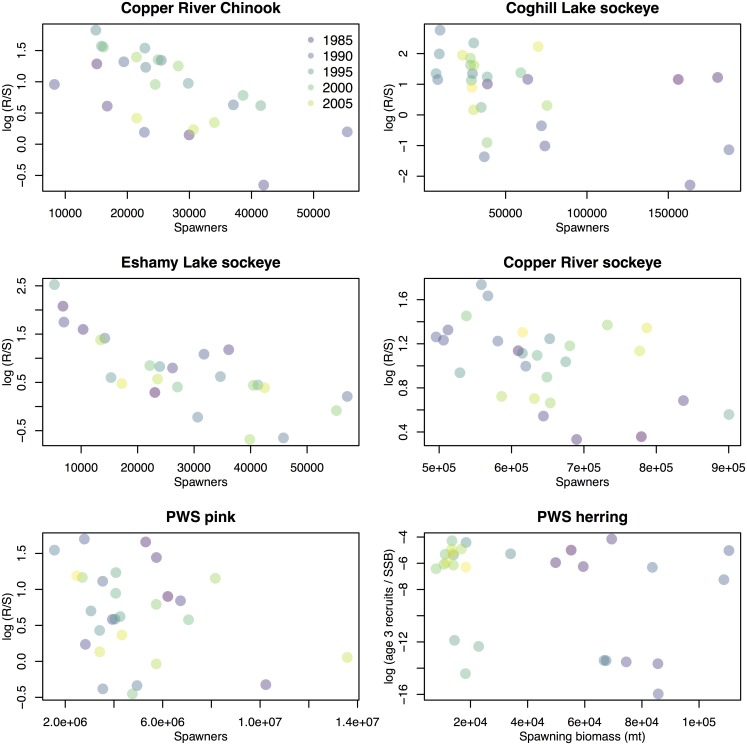
Relationships between spawners (salmon) or spawning stock biomass (herring, in metric tons) and recruits-per-spawner. Raw data are shown for the years included in our analysis, with each year assigned a unique color. R = recruits, S = spawners, SSB = spawning stock biomass, age-3 recruits = millions of mature and immature age-3 herring, and PWS.

To evaluate the hypothesis about intraspecific density dependence, we fit null models with constant productivity to time series for each species, and compared results to those of models that included spawners (or for herring, spawning biomass) in a Ricker stock-recruit relationship (Figs 2–4).

#### Hypothesis 2: Population productivity was negatively impacted by the oil spill

**Fig 5:**
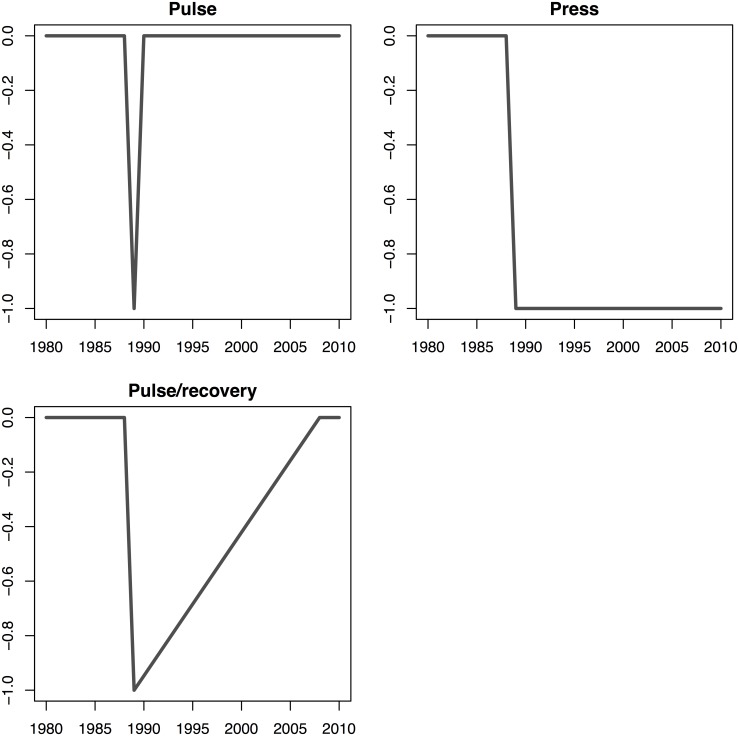
Models of potential impacts (pulse, press, and pulse/recovery) associated with the *Exxon Valdez* oil spill. Impacts here are shown for a time lag of 0, however lag-1 and lag-2 equivalents were also considered as predictors in our models.

To model the potential negative impact of the EVOS event on productivity, we constructed three alternate forms of the impact: a pulse perturbation (the impact of the event lasted one year), a press perturbation (EVOS decreased the long-term mean productivity), and a pulse perturbation followed by a gradual 20-year recovery (length chosen to correspond to a lengthy recovery but fit within the ~25 years of available data, Fig 5). For the herring and salmon species in our analysis, we also included the impacts of the EVOS event with a lag of 0, 1, and 2. All three lags were examined for herring, as spawners, eggs, and larvae may have been immediately impacted in 1989 and juveniles residing in nearshore areas from age 0 to 2 [56] may have been exposed to oil. To model the potential effect of EVOS on salmon species spawning in 1989, we did not lag the indicator covariates. To account for species that may have been exposed to the spill as juveniles, we also considered versions of the EVOS impacts lagged by 1–2 years. For example, species that migrate to the ocean a year after spawning (pink and chum salmon) would have been exposed as 1-year olds, so we allowed the EVOS perturbation to affect the productivity of fish spawning in 1988. Similarly, for species that generally migrate to the ocean as 2-year olds (Chinook, sockeye salmon), we allowed the EVOS perturbation to affect the productivity of fish spawning in 1987.

#### Hypothesis 3: Productivity has been affected by environmental variability

Our third hypothesis involved evaluating data support for effects of changing environmental conditions on herring and salmon productivity. Climate shifts have been suggested as drivers for both salmon and forage fish such as herring [25,57].

For all species, we considered Royer’s annual index of freshwater discharge near Seward [58], because freshwater input has been identified as a potential bottom-up forcing mechanism determining the timing and abundance of zooplankton blooms [59]. For salmon, we constructed species-specific indices of sea surface temperature (SST) and upwelling, depending on life history information and previous work [29,60,61]. For sockeye, we included Jan–Apr SST with a 2-year lag, and the average upwelling from both the winter before and after outmigration (winter defined as Oct–Mar). For pink salmon, there is more uncertainty about whether climate has stronger influences on adult or juveniles, so we included average SST both in the year and season of spawning and the first year in the ocean, as well as upwelling indices in winter (Oct–Mar) and spring (Mar–May) [60]. Because of similar uncertainty with respect to Chinook salmon, we included SST in both the first and second years of ocean life and upwelling indices in both winter and summer (May–Sept) in the first and second years in the ocean. For herring, we considered winter SST (Nov–Mar) immediately before and 1 year prior to spawning, and summer upwelling (May–Sept) 1 and 2 years before spawning [62].

#### Hypothesis 4: Productivity has been shaped by intra- and interspecific interactions among juvenile fish

One of the ecological drivers that may explain trends in herring and salmon productivity (Figs 3 and 4) may be intra- or inter-specific competition as juveniles. Recent trends in hatchery releases in PWS have been dominated by chum and pink salmon (S1 Fig). Research in other regions has suggested that pink salmon may have a competitive advantage over other species, negatively impacting other species’ growth and survival [63–65]. Similarly, interspecific effects of pink salmon on juvenile herring have been hypothesized in PWS [22].

We examined evidence of relationships between productivity and juvenile interactions for herring and the five PWS salmon stocks in our analysis by including time series of hatchery releases of dominant species (pink and chum salmon). For instance, with herring as a response, one hypothesis might be that hatchery pink or chum salmon compete with juvenile herring (age 1). Given the available data, we used hatchery releases in year *t* as a predictor of productivity in year *t-*1 (e.g. hatchery salmon from brood year 1980 would be 1 in 1981 and compete with herring in that year).

#### Hypothesis 5: Predation and adult competition (intra- and inter-specific) has impacted productivity

As our fifth hypothesis, we evaluated support for predation and competition by adults on juveniles of the same or different species and support for predation on herring by humpback whales. For example, predation and competition from returning adult salmon may directly affect juvenile herring and salmon and their prey [38,41]. As a proxy for adult predation on/competition with juveniles, we used estimates of total returning salmon abundance as covariates in our model [54,55]. We further stratified returning pink and chum salmon into wild and hatchery components to evaluate whether either component, or the combined run size, appeared to impact outmigrating juvenile salmon through predation or competition. Examples of these effects included using adult salmon (pink, chum, coho *O*. *kisutch*) returning in year *t* as a predictor of the brood year production from year *t*-1 in the herring models (e.g. herring produced by spawners in 1980 would have been age 1 in 1981, and subject to predation and competition from returning adult salmon that year). For herring, we also included PWS humpback whale abundance [43] as an additional covariate, as they have increased in number since 1970 and may be responsible for additional mortality in other regions [66].

### Statistical analysis

For models of fish recruitment, we assumed that the herring and salmon stock-recruit relationship followed a Ricker model [67]. This model has been widely used in fisheries, because it allows a flexible parameterization but can also be linearized [68]. This stock-recruit model can be written as log(*R/S*)_*t*_ = *a* + *bS*_*t*_ + *cX*_*t*_ + *v*_*t*_, where *a* represents maximum per capita (abundance or biomass) productivity or growth rate of the population, *b* is the negative effect of density dependence, *X*_*t*_ are optional time-varying covariates (e.g. SST, upwelling), *c* represents coefficients linking those covariates to productivity, and *v*_*t*_ represents residual error, assumed to be *v*_*t*_ ~ *Normal*(0, *σ*). Additional models, including dynamic linear models, were also explored. Parameter estimation and model selection was conducted in a maximum likelihood framework, using the MARSS package in R [69,70]. To evaluate the data support for various hypotheses described above, we used the small sample version of Akaike’s Information Criterion (AICc) [22,71]. Code and data to replicate these calculations, as well as the model selection described above, and additional detail is provided: https://github.com/NCEAS/pfx-covariation-pws.

## Results

We found variable support for intraspecific density dependence (Hypothesis 1) in herring and salmon populations in PWS. Herring, Chinook and sockeye (Eshamy Lake and Copper River populations) exhibited strong evidence of increasing productivity at lower densities (Table 1, S1 Table), and pink salmon showed little support for the density dependent model, suggesting that variation may be better explained by other covariates (or that pink salmon escapements have been below thresholds needed to induce density dependence). For the sockeye populations in our analysis, the best model allowed the strength of density dependence to vary by population,(Figs 2–4, S1 Table).

**Table 1:** Table of delta-AIC values used for model selection (S1–S5 Tables include raw values).

Model	Pink	Chinook	Sockeye	Herring
Null (productivity constant)	**0**	20.707	25.896	24.715
1 Ricker 'b' estimated	**0.113**	10.689	21.405	6.439
Ricker 'b' varies by population			10.581	
**EVOS**				
EVOS pulse (lag 0)	2.858	13.644	11.087	7.638
EVOS press (lag 0)	1.624	1.817	12.817	9.296
EVOS pulse/recovery (lag 0)	1.205	**0**	13.179	9.095
EVOS pulse (lag 1)	**0.98**			7.481
EVOS press (lag 1)	3.052			8.516
EVOS pulse/recovery (lag 1)	2.867			7.946
EVOS pulse (lag 2)	2.9	10.877	12.395	7.72
EVOS press (lag 2)	2.793	7.926	13.28	6.071
EVOS pulse/recovery (lag 2)	2.546	7.732	13.217	5.327
**Environmental**				
SST (lag 0)	2.826	12.235		2.915
SST (lag 1)	**0.423**	13.91		8.684
SST (lag 2)			12.875	
Upwelling winter (lag 1)	3.104	11.469	13.018	
Upwelling winter (lag 2)	3.085	13.425	13.202	
Upwelling spring (lag 1)	3.088			
Upwelling spring (lag 2)	2.664			
Upwelling summer (lag 1)		8.887		7.32
Upwelling summer (lag 2)		13.315		9.195
Freshwater discharge (lag 0)	2.346	13.327	12.582	**0**
Freshwater discharge (lag 1)	2.459	12.405	13.435	9.448
**Juvenile competition**				
Hatchery pink releases	**0.304**	8.311	13.14	7.965
Hatchery chum releases	2.764	11.195	13.039	9.243
**Competition and predation**				
Wild chum	3.071	12.778	12.518	8.54
Wild pink	2.975	9.867	11.872	6.099
Hatchery chum	3.095	6.464	12.93	5.352
Hatchery pink	1.488	12.391	**0**	9.093
Total pink run	2.106	13.84	3.5	8.105
Humpback whales				7.851

Models with the most support are indicated with a zero; all models within one log-likelihood unit highlighted in bold.

We found little support for any negative impact of the EVOS (Hypothesis 2) on long term productivity in these populations (Table 1, S2 Table). Chinook salmon supported the inclusion of the EVOS covariate in explaining variation in productivity relative to the models that only included density dependence (Table 1), but the estimated impact of EVOS was slightly positive and opposite of what we might expect from other studies [16]. Coefficients for these impacts and all hypotheses are included online, https://github.com/NCEAS/pfx-covariation-pws.

**Fig 6:**
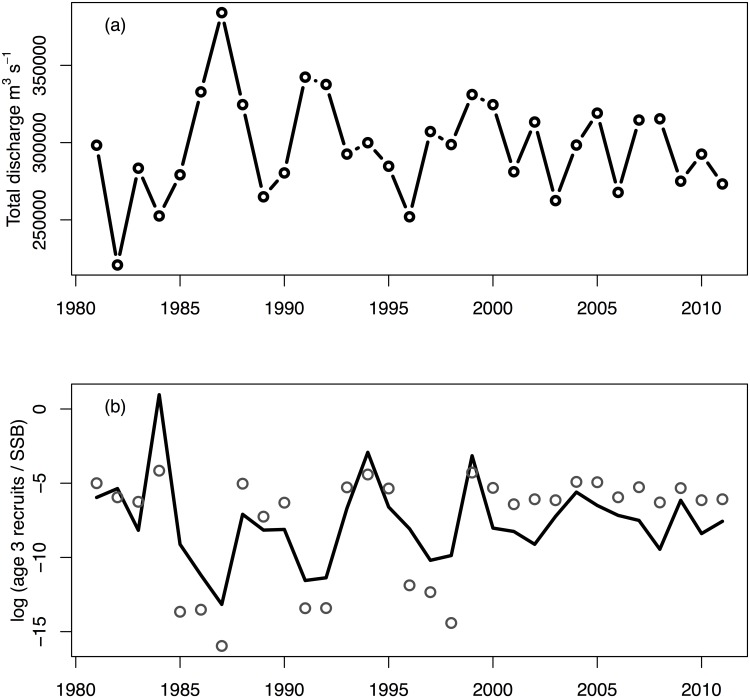
Gulf of Alaska freshwater discharge (Royer 1982, IMS 2016) as a driver of Pacific herring productivity. Shown are (a) the total freshwater discharge (m^3^ s^-1^) and (b) log of observed age-3 recruits per spawning biomass (mt)—log(recruits/SSB)—in grey circles, and the model predicted log(recruits/SSB) using freshwater discharge as a covariate (R^2^ = 0.55). High discharge events correspond to reduced productivity (fewer recruits to the population as three year olds). For historical reference, the discharge time series starting in 1931 is shown in S2 Fig. R = millions of mature and immature age-3 herring, SSB = spawning stock biomass in metric tons.

The strongest relationship between the environmental covariates (Hypothesis 3) we examined and productivity was the estimated effect of freshwater discharge on herring (Table 1, S3 Table; Fig 6). The estimated productivity was lower than average in years of high discharge. Discharge into the Gulf of Alaska was episodic both before and after the EVOS event, and periods of high discharge generally coincided with three multi-year herring productivity failures (Fig 6; 1985–1987, 1991–1992, and 1996–1998). Our results showed less evidence for environmental drivers of salmon productivity; although, summer and winter upwelling were identified as predictors of Chinook and sockeye salmon productivity, respectively (Table 1, S3 Table). In both cases, however, models with environmental covariates performed worse when compared to all hypotheses (Table 1).

In evaluating hypotheses about effects of juvenile-juvenile competition (Hypothesis 4), we found little support for linking hatchery or wild pink or chum salmon to declining productivity of examined species (S4 Table). Including hatchery releases slightly worsened the fit of our model of wild pink salmon productivity, but was within 1 log likelihood of the best model (constant productivity). The effect of hatchery pink salmon releases was estimated to be slightly positive on juvenile Chinook salmon. Statistically, the inclusion of this predictor was an improvement over the null model for Chinook salmon (S4 Table); however, there was no support in including it in the model that also included the EVOS pulse/recovery impact.

**Fig 7:**
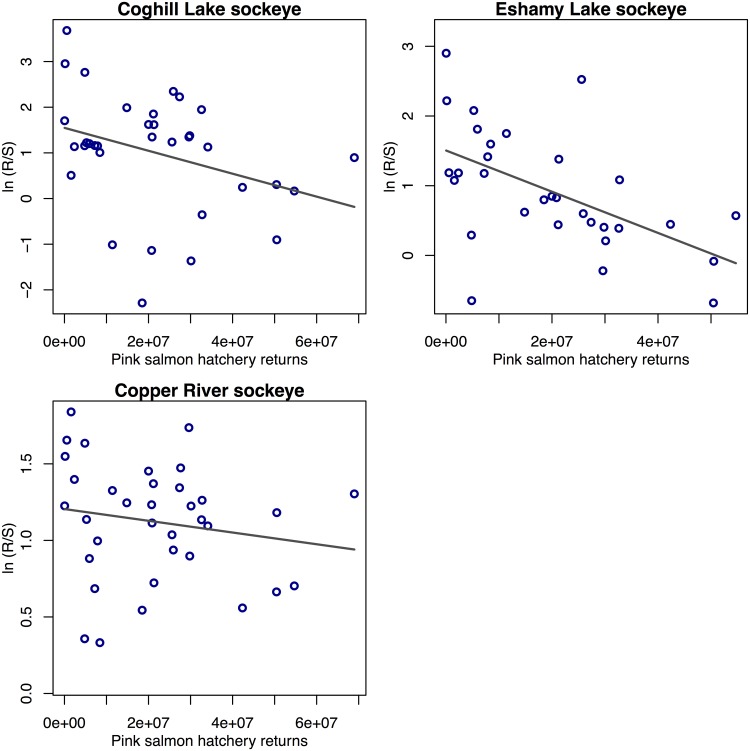
Sockeye salmon productivity, log(R/S), vs. total hatchery pink salmon returns to PWS. Black lines are best-fit lines from linear regressions fit separately to each time series. Note that the multivariate time-series method we used for the analysis is a different approach than the simple linear relationships shown here. Similar trends in the residuals also exist after the effect of spawning abundance is removed (see S3 Fig).

We found a negative relationship between adult hatchery pink salmon returns on sockeye salmon productivity, supporting the predation and adult competition hypothesis (Hypothesis 5) (Table 1, Fig 7, S5 Table, and S3 Fig); however, this effect was not found for herring, Chinook, or wild pink salmon. The lag-2 model of hatchery returns was most supported, suggesting that adult hatchery pink salmon returning in year *Y* had a negative effect on the sockeye recruitment of brood year *Y*-2 (the 2 year lag a result of sockeye rearing in freshwater for 2 years before emigrating to the ocean). To understand the magnitude of these estimated hatchery pink salmon effects, we used the mean number of pink hatchery returns over the time series (2.5e+07) and mean log-productivity across the 3 sockeye populations in our analysis (0.87) to calculate the effect size of a 10% increase in pink salmon returns; this translates to log(R/S) declining to 0.938 of the status quo. For wild pink salmon productivity, including predation and competition from hatchery pink salmon worsened the fit of the models slightly (S5 Table). We found a slight improvement in models of herring productivity when interactions with adult wild pink salmon or hatchery chum salmon were included, although these effects were contrasting, with a negative effect of chum and a positive effect of wild pink salmon.

## Discussion

The short- and long-term impacts of the *Exxon Valdez* oil spill, and coincident changes in climate and the ecological community of Prince William Sound have remained controversial, even nearly three decades after EVOS [12,13,23]. Our results largely support the idea that longer term changes in herring and salmon productivity in PWS have been affected by multiple processes, including negative effects of spawner density dependence (for herring, Chinook, and sockeye), changing environmental conditions (freshwater discharge for herring), and interspecific effects such as negative impact of adult hatchery pink salmon on wild sockeye salmon productivity. We also note that in order to accommodate the inclusion of multiple species, our analysis of productivity begins in 1981, several years after the onset of hatchery production in PWS [35] and the 1976–77 regime shift [28].

We found no evidence supporting a negative EVOS impact on herring, sockeye salmon, or pink salmon productivity, and weak evidence of a slightly positive EVOS signal (in the press-recovery model) on Copper River Chinook salmon productivity. It is unclear how EVOS may have impacted Chinook salmon positively. This result may be spurious, or Chinook salmon in particular may have benefitted from the substantial reduction in some predators; including the deaths of as many as several hundred thousand seabirds [72] and severe losses to pods of killer whales (*Orcinus orca*) [73] as a direct result of EVOS. Acute exposure to oil has known impacts on hatchery and wild fish [16], when measured at the individual level in a controlled environment. But when examining productivity at a population level, this may be much more difficult to detect, because the exposure of individual fish to oil is unknown, recruitment is highly variable, and recruitment and spawning numbers or biomass may change together. Further, the species included in our analysis exhibit life history variation that may help further buffer them from perturbations (as a ‘portfolio’ effect; [74]). For example, Chinook, sockeye, and chum salmon, have variation in age at maturity such that returns from a single brood year are dispersed across several years [75].

Though we found no evidence relating herring productivity to EVOS, or most climate drivers, we did find evidence of a strong negative correlation between herring productivity and freshwater discharge into the Gulf of Alaska. This finding suggests that herring survival may be vulnerable to changing climate conditions which may be affecting herring survival via multiple pathways. Over the past 40 years, the northern Gulf of Alaska has undergone a general warming and freshening in the upper 100 m of the water column, and an increase in salinity in depths between 100–200 m. This suggests that vertical stratification of the upper water column in the Gulf of Alaska has increased substantially over this time frame [30,33]. A second effect of warming conditions may be changing amounts of rain and snowpack melted, as well as the timing of the spring discharge [30]. Other studies have found support for increased freshwater discharge suppressing phytoplankton and favoring microbial production [76]. Though the relative importance of these pathways on plankton biomass is not known, the shift in timing and/or decreased primary production related to increasing water temperatures and water column stability, or increased freshwater inputs may be one of many factors that have kept herring abundances in the north-central Gulf of Alaska low over the past 25 years.

For the majority of the interspecific interactions we examined, including juvenile-juvenile competition, or adult competition and predation, we found little data support. All sockeye salmon stocks examined exhibited a downward trend in productivity with increasing PWS hatchery pink salmon returns (Fig 7, S3 Fig). While there was considerable variation in sockeye salmon productivity across the low- and mid-range of hatchery returns (0–30 million), productivity was particularly impacted at higher levels of hatchery returns. Pink salmon have been found to negatively affect sockeye salmon productivity and growth from British Columbia and Southeast Alaska [63,64], Bristol Bay [65], Kodiak [77,78], and Russia [79]. Pink and sockeye salmon compete in the marine environment due to a high degree of similarity in diets [40,80,81], including similarities in diets of adult pink salmon and juvenile sockeye salmon [82,83]. Our analysis was primary designed to test drivers in the nearshore environment, which is why we stopped at a lag of 2 (brood) years—when the majority of juvenile sockeye salmon outmigrate from the nearshore environment as adult pink salmon are returning to spawn. We do not know if possible deleterious interactions between hatchery pink salmon and wild sockeye salmon in this study are from predation or competition, or whether they occur in nearshore or offshore areas. Pink salmon feeding may cause a general depletion of prey availability [38] that could impact sockeye salmon without tight spatial overlap of these two species. In this regard, the apparent impact to sockeye productivity may reflect a general increase in pink salmon abundance across the NE Pacific rather than increased abundance of hatchery pink salmon to PWS in particular. However, adult pink salmon are known to feed on a broad diversity of prey items within PWS prior to spawning, including a variety of zooplankton [41]; and therefore have the potential to compete with juvenile sockeye salmon in PWS for the same prey. For example, Martinson et al. [77] showed decreased growth of sockeye salmon outmigrating from the Karluk River (Kodiak, AK) during years when large numbers of adult pink salmon returned to the same area. Competitive interactions in nearshore and offshore environments deserve greater attention in future research in the face of general increase in the abundance of pink salmon in the North Pacific [38,84,85].

Although our results did not show common drivers for salmon and herring productivity during the timespan of our analysis (1981–2014), it is possible that other drivers—rooted in the 1976–77 and 1989 regime shifts [28,29,86]—resulted in the similar trends in salmon and herring spawning populations in PWS during a relatively narrow timespan. For PWS herring, the large adult spawning biomass of the 1980s–early 1990s can be traced to strong recruitment from the 1976, 1984, and 1988 year classes, which has not occurred during more recent years [87–89]. The three salmon stocks located inside PWS (wild: pink salmon, Cogill Lake and Eshamy Lake sockeye) exhibited record high levels of productivity and increased abundance for brood years that entered the marine environment immediately following the 1976–77 regime shift (Fig 2). For wild pink salmon, record high return-per-spawner (R/S) and six of the top ten total returns occurred from the 1977–1988 brood years. For the Coghill Lake sockeye salmon population, the 1976 and 1977 brood years had by far the highest R/S on record and four of the top five total returns originated from brood years 1976–1984. For the Eshamy Lake sockeye salmon population, record R/S occurred for brood years 1974 and 1975 (first marine years 1976 and 1977) and all five of the largest historical brood-year returns occurred before 1988 (https://github.com/NCEAS/pfx-covariation-pws). Two stocks in the PWS region not included in our productivity analysis (wild PWS chum and Unakwik District sockeye salmon, S4 and S5 Figs) also experienced dramatic increases in abundance (wild chum salmon) and harvest (Unakwik sockeye) from brood years following the 1976–77 regime shift, but declined by the late 1980s. Thus, populations in PWS showed dramatic increases in abundance by 1979 (pink salmon) or early 1980s (herring, chum and sockeye salmon) with declines by the late 1980s (sockeye salmon) or early 1990s (wild pink and chum salmon, herring). As noted by others (e.g., [17]), declines in abundance for wild salmon occurred for cohorts of species (pink, sockeye, and chum salmon) that were not directly exposed to EVOS at either the adult or juvenile stages. For example, low returns of wild pink salmon in 1992 and 1993, Coghill and Eshamy sockeye salmon during 1990, and wild chum salmon beginning in 1991 (S4 Fig, https://github.com/NCEAS/pfx-covariation-pws).

Changes in herring and salmon populations in PWS between the late 1970s and early 1990s came about at a time of large-scale changes for other species groups in the Gulf of Alaska, including declines in populations of forage fish, birds, and marine mammals; and increased abundances of gadids—walleye pollock (*Gadus chalcogrammus*) in particular [25,57,90–93]. For PWS, a directed commercial trawl fishery for walleye pollock was initiated in 1995 after observations of substantial pollock biomass with acoustics [94], and annual harvests of pollock have ranged from approximately 1000–3000 metric tons since [94,95]. Studies conducted in the late 1980s and early 1990s showed that walleye pollock and other gadids had become a significant component in the diets of birds in PWS and the Gulf of Alaska [72,93] that there is substantial dietary and spatial overlap between walleye pollock and herring [92,96]. Like other possible factors that may influence salmon and herring populations, walleye pollock were not considered in our analyses due to the absence of annual population-level estimates for PWS. However, given the dietary overlap and the increased abundance of walleye pollock around the time of the declining herring populations in PWS, we consider the interactions between walleye pollock and herring in PWS to be deserving of additional study.

In contrast to the PWS salmon and herring stocks described above, stocks of sockeye and Chinook salmon from the adjacent Copper River system did not experience a concomitant decline in abundance in the late 1980s or early 1990s (Fig 2). Total returns of Copper River sockeye have remained at historically high levels from the early 1980s to the time of this writing [54,55]; and only since 2008 have returns of Copper River Chinook declined, possibly in association with a broad-scale phenomena that have impacted this species across Alaska [97]. These differences in population trends indicate that, compared with PWS, alternate processes may influence salmon populations originating from the Copper River area.

## Conclusions

The five major hypotheses examined here cover potentially important drivers for salmon and herring, but the lack of support for many of these predictors suggest that other factors may also be important (e.g., [17]). For example, we did not include covariates that only existed for portions of the time series, such as disease. Disease has been proposed as one mechanism for explaining declines in herring abundance in PWS [98–100]. The PWS herring disease data (1994–present) starts after EVOS and other climatic perturbations and therefore cannot be used to assess the decline of herring during 1992–93. We also did not evaluate support for long term effects of human resource use, including commercial fishing. Fishing practices may interact with climate variation [101], or make stocks more vulnerable to population collapse [102].

The contrast between recent studies that have demonstrated negative toxicity of oil on fishes and our results indicating little support for an effect at the population level also suggests a need for better data on the exposure of individual fish to oil after spills occur. Incardona et al. [16] suggested a mechanism by which detrimental effects could result from low toxicity 7–9 months after exposure, fine scale sampling of individual exposure rates immediately following a spill could be combined with intensive spatiotemporal histology sampling in the years that follow.

Better understanding the processes responsible for changing environmental drivers on marine fish like salmon and herring is essential, particularly when these processes link terrestrial and aquatic ecosystems, and are affected by variables like freshwater discharge, which is sensitive to effects of climate change [103]. Looking at the entire time series of freshwater discharge into the Gulf of Alaska (S2 Fig), the variability appears to be dampening over time. The mechanism responsible for this dampening is unknown, but it may be partially responsible for less common low discharge events (coincident with herring recruitment pulses). Though herring recruitment data aren’t available for much of the 20^th^ century, the mid-1930s may have been an extremely productive period for herring because of discharge patterns during that time (the most negative discharge anomaly in the mid-1930s, S2 Fig, was immediately followed by the highest herring landings ever recorded; [88]). Just as the previous analyses have evaluated synchrony in herring populations in the NE Pacific Ocean [104], it is important to understand how drivers like freshwater discharge vary spatially. Like many salmon populations in the NE Pacific, herring population dynamics may be synchronized through time and may be shaped in part by external climate drivers. It remains unclear the degree to which asynchrony between herring in the Gulf of Alaska or elsewhere may exhibit a portfolio effect [105,106] and buffer the larger metapopulation from future perturbations.

## Supporting information

S1 Fig Hatchery release trends for coho, sockeye, chum, and pink salmon, 1979–2014. (TIFF)

S2 Fig Historic freshwater discharge into Prince William Sound, 1931–2010 (Royer 1982, IMS 2016). The dashed horizontal line represents the mean, and the dashed vertical lines represent the time period included in our analyses.
(TIFF)

S3 Fig Residuals from a simple Ricker stock-recruit model fit separately to each population. Sockeye time series versus year and total pink salmon hatchery returns (neither covariate included in this model). Using the model selection described in main text and a model that integrates all three time series in the same analysis, the model with the inclusion of pink salmon returns is supported because of the negative trend in residuals (particularly for Eshamy and Coghill).
(TIFF)

S4 Fig Total estimated run size of wild chum in Prince William Sound. (TIFF)

S5 Fig Total harvest of Unakwik District sockeye salmon. (TIFF)

S1 Table Detailed results for models that only include density dependence. Table of model selection values (AICc) comparing null models (constant productivity, or log(R/S) independent of spawners) to models that estimated density dependence via the Ricker stock-recruitment relationship. For each species, the best model and all models within 1 log-likelihood unit are highlighted in bold (the best model only being defined for this particular table—all results are included in Table 1).
(DOCX)

S2 Table Detailed results for models that only include effects of EVOS. Table of model selection values (AICc) comparing models without covariates (i.e. models presented in S1 Table) to models that also estimate an impact of the EVOS event (pulse, press, pulse/recovery with various lags). All models that include an EVOS impact also include density dependence (the sockeye models with EVOS allowed density dependence to vary by population). For each species, the best model and all models within 1 log-likelihood unit are highlighted in bold (the best model only being defined for this particular table—all results are included in Table 1). Lag-1 impacts were not considered on Chinook and sockeye, as these species generally migrate to the ocean in their second year of life.
(DOCX)

S3 Table Detailed results for models that only include environmental covariates. Table of model selection values (AICc) comparing models without covariates (i.e. models presented in S1 Table) to models that also estimate an impact of environmental effects. All models that include environmental predictors also include density dependence (the sockeye models with environmental effects allowed density dependence to vary by population). For each species, the best model and all models within 1 log-likelihood unit are highlighted in bold (the best model only being defined for this particular table—all results are included in Table 1). Additional details included online, https://github.com/NCEAS/pfx-covariation-pws.
(DOCX)

S4 Table Detailed results for models that only include effects of juvenile competition. Table of model selection values (AICc) comparing models without covariates (i.e. models presented in S1 Table) to models that also estimate an impact of juvenile competition. All models with juvenile competition included also include density dependence (the sockeye models with juvenile competition allowed density dependence to vary by population). For each species, the best model and all models within 1 log-likelihood unit are highlighted in bold (the best model only being defined for this particular table—all results are included in Table 1).
(DOCX)

S5 Table Detailed results for models that only include effects of predation and adult competition. Table of model selection values (AICc) comparing models without covariates (i.e. models presented in S1 Table) to models that also estimate an impact of predation or adult competition on wild salmon productivity. All models with predation or adult competition included also include density dependence (the sockeye models with predation or adult competition allowed density dependence to vary by population). For each species, the best model and all models within 1 log-likelihood unit are highlighted in bold (the best model only being defined for this particular table—all results are included in Table 1). All salmon models used the estimated total run size of adult salmon.
(DOCX)
