# Correction: Evaluating signals of oil spill impacts, climate, and species interactions in Pacific herring and Pacific salmon populations in Prince William Sound and Copper River, Alaska

**DOI:** 10.1371/journal.pone.0197873

**Published:** 2018-05-22

**Authors:** Eric J. Ward, Milo Adkison, Jessica Couture, Sherri C. Dressel, Michael A. Litzow, Steve Moffitt, Tammy Hoem Neher, John Trochta, Rich Brenner

There are errors in Table 1, S1–S5 Tables, and Fig 6. A coding error uses recruits per spawner as a covariate instead of using spawning biomass as a predictor of ln (recruits per spawner). The correct code produces greater uncertainty in mechanisms responsible for variability in herring recruitment. Please see the corrected Table 1, S1–S5 Tables, and Fig 6 here.

**Fig 6:**
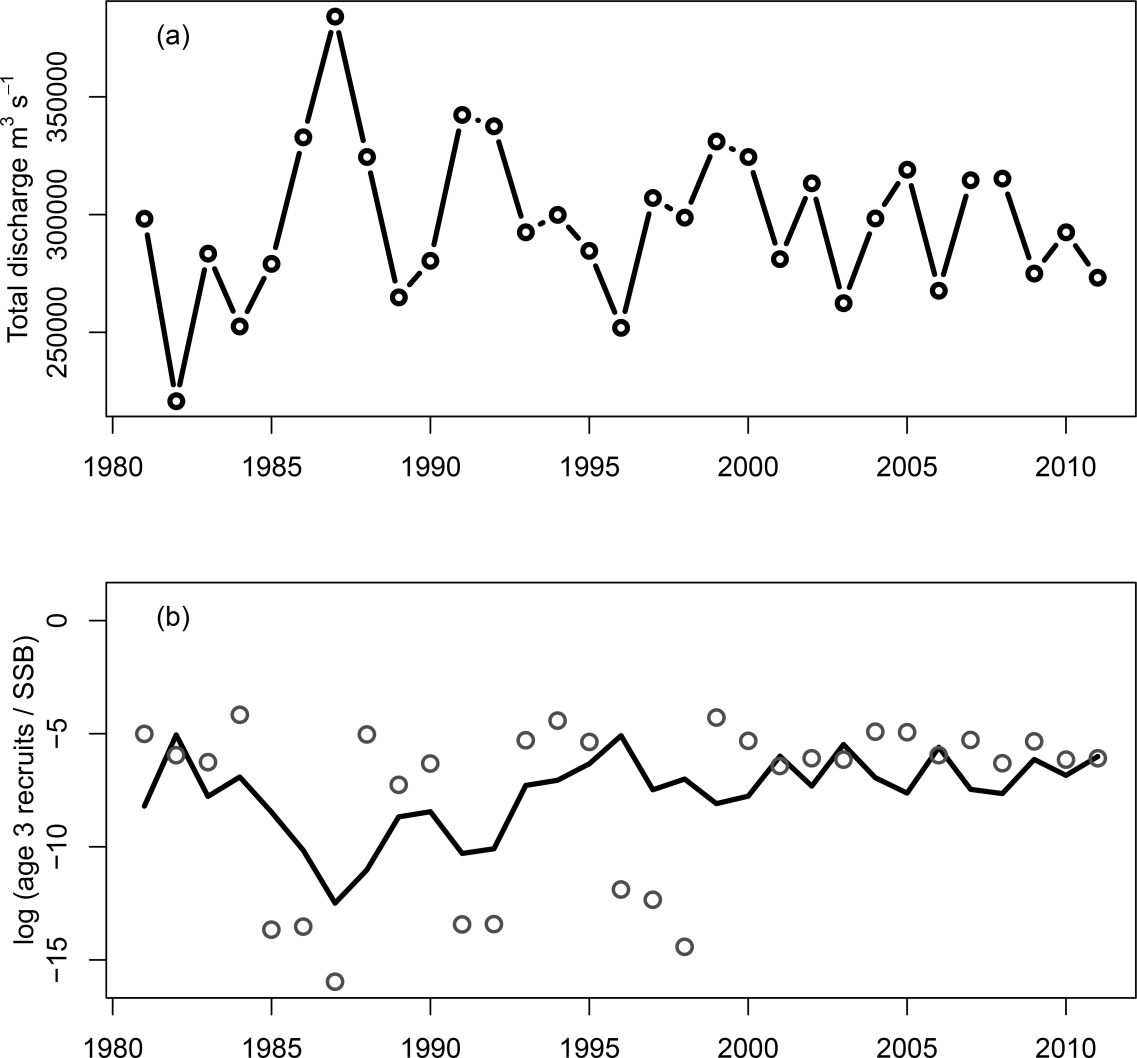
Gulf of Alaska freshwater discharge (Royer 1982, IMS 2016) as a driver of Pacific herring productivity. Shown are (a) the total freshwater discharge (m^3^ s-1) and (b) log of observed age-3 recruits per spawning biomass (mt)—log(recruits/SSB)—in grey circles, and the model predicted log(recruits/SSB) using freshwater discharge as a covariate (R^2^ = 0.55). High discharge events correspond to reduced productivity (fewer recruits to the population as three year olds). For historical reference, the discharge time series starting in 1931 is shown in S2 Fig. R = millions of mature and immature age-3 herring, SSB = spawning stock biomass in metric tons.

**Table 1:** Table of delta-AIC values used for model selection (S1–S5 Tables include raw values).

Model	Pink	Chinook	Sockeye	Herring
Null (productivity constant)	**0**	20.707	25.896	**0.692**
1 Ricker 'b' estimated	**0.113**	10.689	21.405	2.23
Ricker 'b' varies by population			10.581	
**EVOS**				
EVOS pulse (lag 0)	2.858	13.644	11.087	4.236
EVOS press (lag 0)	1.624	1.817	12.817	4.759
EVOS pulse/recovery (lag 0)	1.205	**0**	13.179	4.393
EVOS pulse (lag 1)	**0.98**			2.027
EVOS press (lag 1)	3.052			4.965
EVOS pulse/recovery (lag 1)	2.867			5.091
EVOS pulse (lag 2)	2.9	10.877	12.395	**0.597**
EVOS press (lag 2)	2.793	7.926	13.28	4.316
EVOS pulse/recovery (lag 2)	2.546	7.732	13.217	3.764
**Environmental**				
SST (lag 0)	2.826	12.235		3.38
SST (lag 1)	**0.423**	13.91		3.288
SST (lag 2)			12.875	
Upwelling winter (lag 1)	3.104	11.469	13.018	
Upwelling winter (lag 2)	3.085	13.425	13.202	
Upwelling spring (lag 1)	3.088			
Upwelling spring (lag 2)	2.664			
Upwelling summer (lag 1)		8.887		4.8
Upwelling summer (lag 2)		13.315		3.91
Freshwater discharge (lag 0)	2.346	13.327	12.582	**0.372**
Freshwater discharge (lag 1)	2.459	12.405	13.435	4.848
**Juvenile competition**				
Hatchery pink releases	**0.304**	8.311	13.14	4.97
Hatchery chum releases	2.764	11.195	13.039	1.425
**Competition and predation**				
Wild chum	3.071	12.778	12.518	4.629
Wild pink	2.975	9.867	11.872	**0**
Hatchery chum	3.095	6.464	12.93	4.172
Hatchery pink	1.488	12.391	**0**	1.609
Total pink run	2.106	13.84	3.5	3.106
Humpback whales				3.949

There is an error in the second sentence of the first paragraph of the Results. The correct sentence is: Chinook and sockeye (Eshamy Lake and Copper River populations) exhibited strong evidence of increasing productivity at lower densities (Table 1, S1 Table), and pink salmon showed little support for the density dependent model, suggesting that variation may be better explained by other covariates (or that pink salmon escapements have been below thresholds needed to induce density dependence).

The following sentence should be included before the final sentence of the second paragraph of the Results: There was also some support for the inclusion of a pulsed EVOS effect in herring recruitment, though this model performed similarly to models with other covariates, or a simpler model without the EVOS effect included.

There is an error in the first sentence of the first paragraph of the Discussion. The correct sentence is:

We found no evidence supporting a negative EVOS impact on sockeye salmon, or pink salmon productivity, weak evidence of a slightly positive EVOS signal (in the press-recovery model) on Copper River Chinook salmon productivity, and weak evidence of a negative pulse effect on herring productivity.

There is an error in the first sentence of the third paragraph of the Discussion. The correct sentence is: In addition to the weak evidence relating herring productivity to EVOS, we also found some evidence of a negative correlation between herring productivity and freshwater discharge into the Gulf of Alaska.

## Supporting information

S1 Table Detailed results for models that only include density dependence. Table of model selection values (AICc) comparing null models (constant productivity, or log(R/S) independent of spawners) to models that estimated density dependence via the Ricker stock-recruitment relationship. For each species, the best model and all models within 1 log-likelihood unit are highlighted in bold (the best model only being defined for this particular table—all results are included in Table 1).
(DOCX)

S2 Table Detailed results for models that only include effects of EVOS. Table of model selection values (AICc) comparing models without covariates (i.e. models presented in S1 Table) to models that also estimate an impact of the EVOS event (pulse, press, pulse/recovery with various lags). All models that include an EVOS impact also include density dependence (the sockeye models with EVOS allowed density dependence to vary by population). For each species, the best model and all models within 1 log-likelihood unit are highlighted in bold (the best model only being defined for this particular table—all results are included in Table 1). Lag-1 impacts were not considered on Chinook and sockeye, as these species generally migrate to the ocean in their second year of life.
(DOCX)

S3 Table Detailed results for models that only include environmental covariates. Table of model selection values (AICc) comparing models without covariates (i.e. models presented in S1 Table) to models that also estimate an impact of environmental effects. All models that include environmental predictors also include density dependence (the sockeye models with environmental effects allowed density dependence to vary by population). For each species, the best model and all models within 1 log-likelihood unit are highlighted in bold (the best model only being defined for this particular table—all results are included in Table 1). Additional details included online, https://github.com/NCEAS/pfx-covariation-pws.
(DOCX)

S4 Table Detailed results for models that only include effects of juvenile competition. Table of model selection values (AICc) comparing models without covariates (i.e. models presented in S1 Table) to models that also estimate an impact of juvenile competition. All models with juvenile competition included also include density dependence (the sockeye models with juvenile competition allowed density dependence to vary by population). For each species, the best model and all models within 1 log-likelihood unit are highlighted in bold (the best model only being defined for this particular table—all results are included in Table 1).
(DOCX)

S5 Table Detailed results for models that only include effects of predation and adult competition. Table of model selection values (AICc) comparing models without covariates (i.e. models presented in S1 Table) to models that also estimate an impact of predation or adult competition on wild salmon productivity. All models with predation or adult competition included also include density dependence (the sockeye models with predation or adult competition allowed density dependence to vary by population). For each species, the best model and all models within 1 log-likelihood unit are highlighted in bold (the best model only being defined for this particular table—all results are included in Table 1). All salmon models used the estimated total run size of adult salmon.
(DOCX)

## References

[1] Ward EJ, Adkison M, Couture J, Dressel SC, Litzow MA, Moffitt S, et al (2017) Evaluating signals of oil spill impacts, climate, and species interactions in Pacific herring and Pacific salmon populations in Prince William Sound and Copper River, Alaska. PLoS ONE 12(3): e0172898 https://doi.org/10.1371/journal.pone.0172898 28296895 10.1371/journal.pone.0172898 PMC5351843

