# Supplementary figures and images for: Evaluating signals of oil spill impacts, climate, and species interactions in Pacific herring and Pacific salmon populations in Prince William Sound and Copper River, Alaska
Source: PLoS One. 2017 Mar 15;12(3):e0172898. doi: 10.1371/journal.pone.0172898 (PMC5351843; doi:10.1371/journal.pone.0172898)

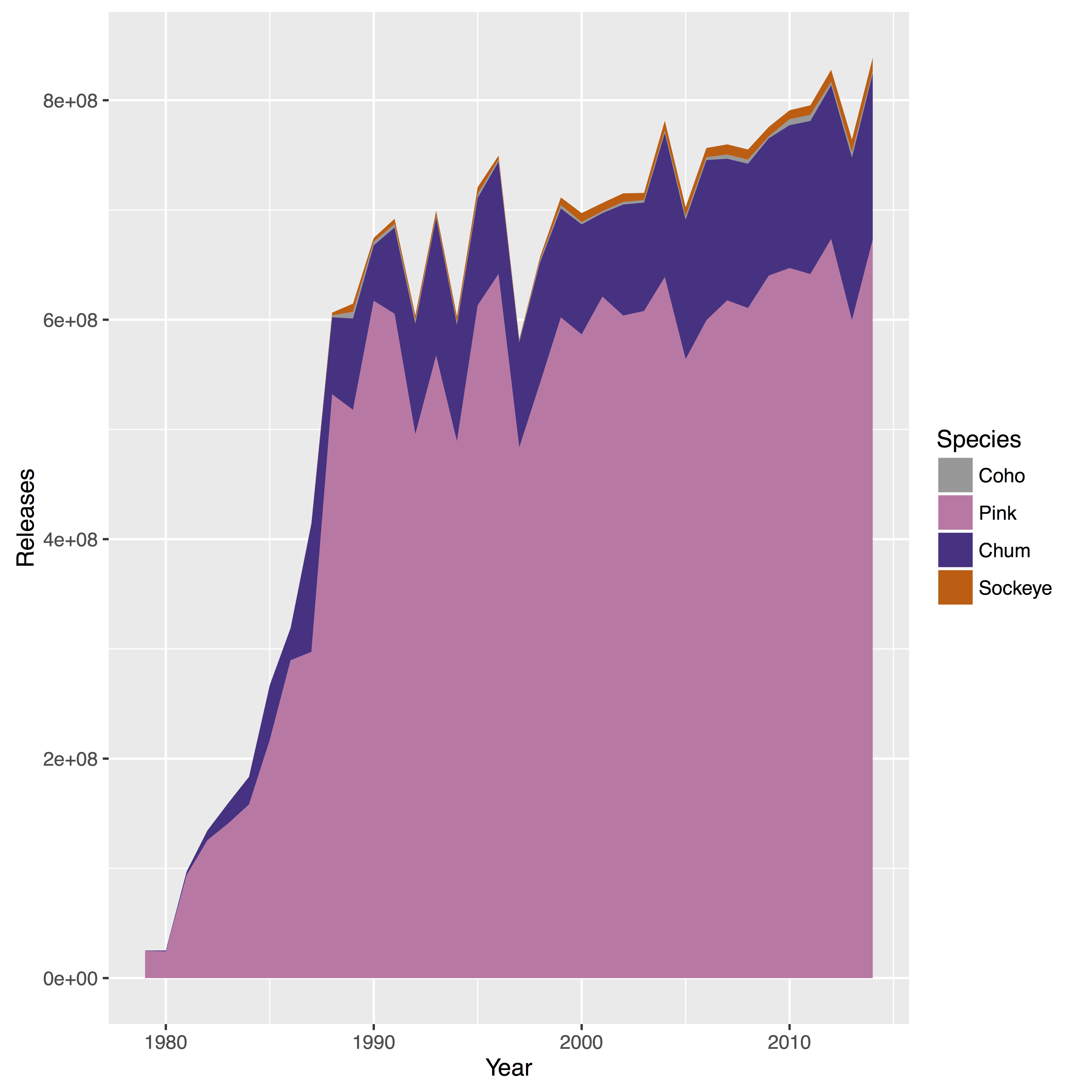

Supplement: S1 Fig — (TIFF) [file pone.0172898.s001.tiff]

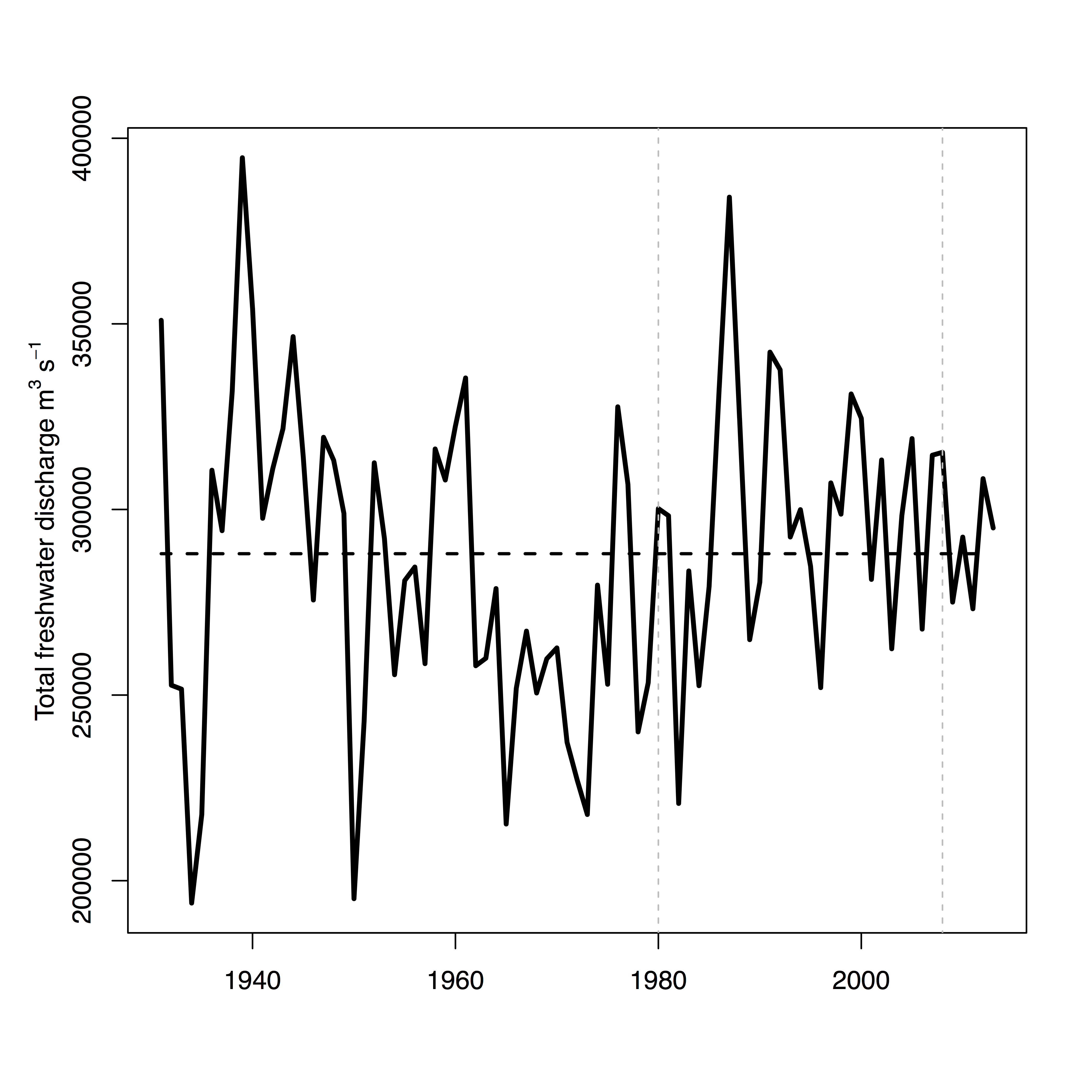

Supplement: S2 Fig — The dashed horizontal line represents the mean, and the dashed vertical lines represent the time period included in our analyses. (TIFF) [file pone.0172898.s002.tiff]

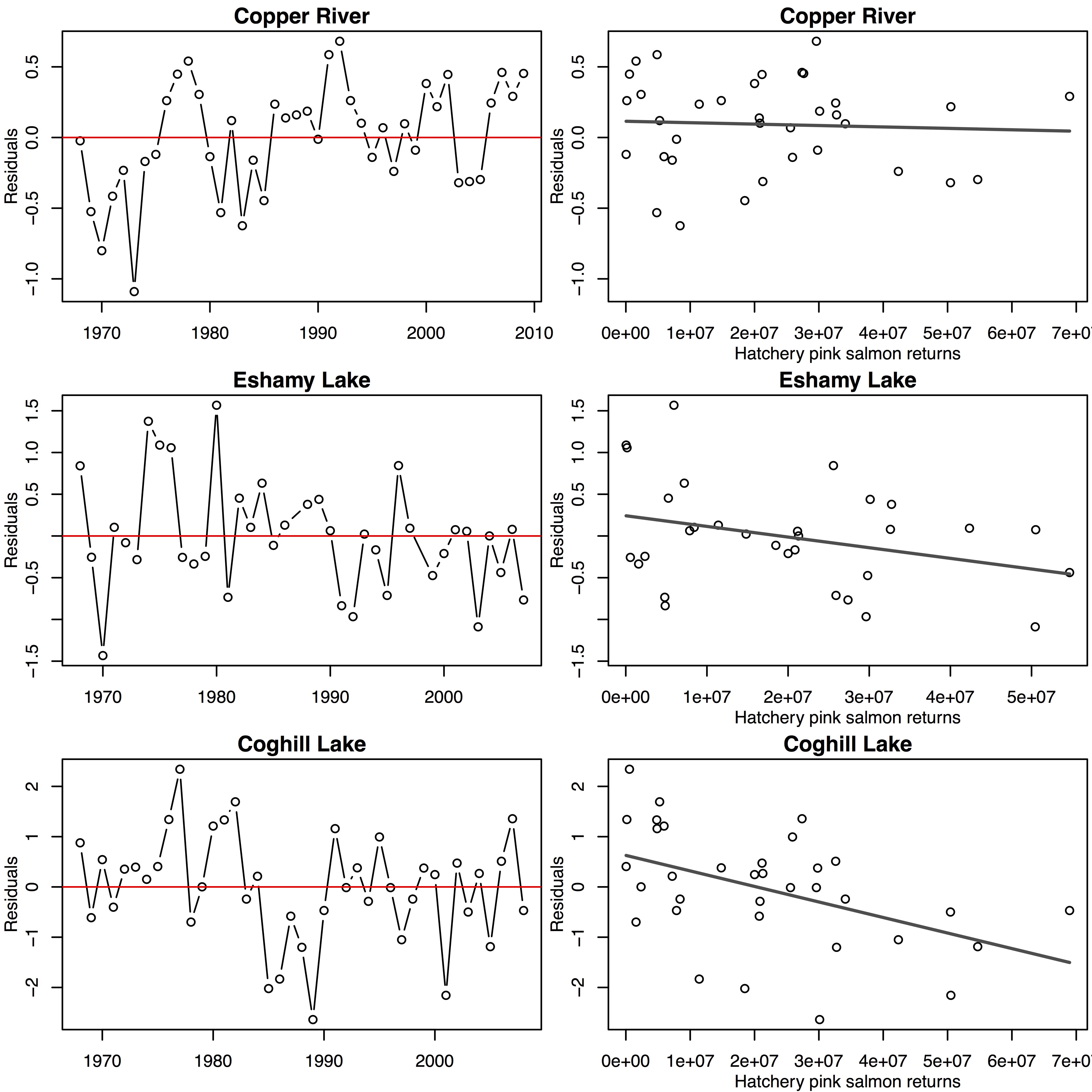

Supplement: S3 Fig — Sockeye time series versus year and total pink salmon hatchery returns (neither covariate included in this model). Using the model selection described in main text and a model that integrates all three time series in the same analysis, the model with the inclusion of pink salmon returns is supported because of the negative trend in residuals (particularly for Eshamy and Coghill). (TIFF) [file pone.0172898.s003.tiff]

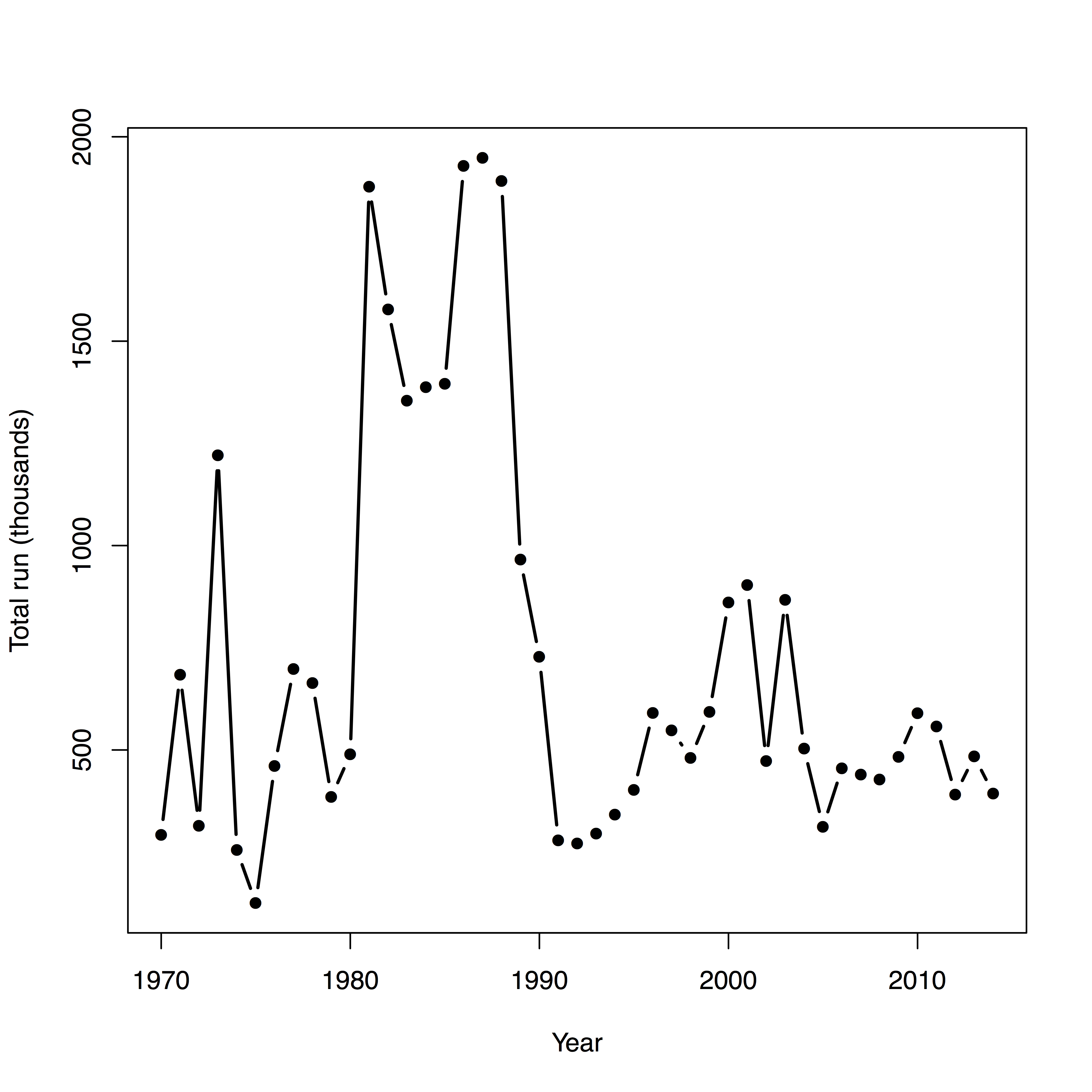

Supplement: S4 Fig — (TIFF) [file pone.0172898.s004.tiff]

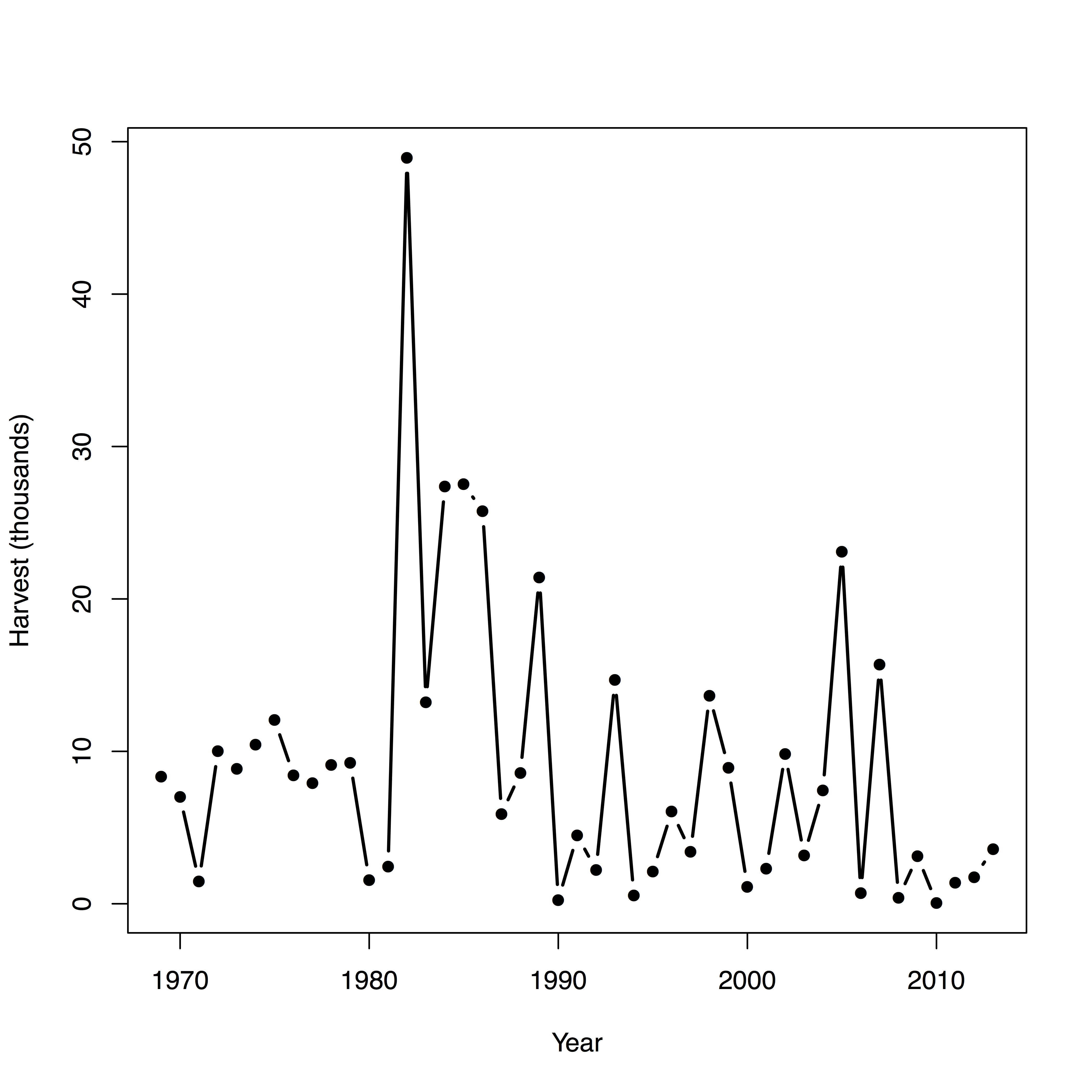

Supplement: S5 Fig — (TIFF) [file pone.0172898.s005.tiff]
