# Supplementary material for: Evaluating signals of oil spill impacts, climate, and species interactions in Pacific herring and Pacific salmon populations in Prince William Sound and Copper River, Alaska
Source: PLoS One. 2017 Mar 15;12(3):e0172898. doi: 10.1371/journal.pone.0172898 (PMC5351843; doi:10.1371/journal.pone.0172898)
Supplement: S2 Table — Table of model selection values (AICc) comparing models without covariates (i.e. models presented in S1 Table) to models that also estimate an impact of the EVOS event (pulse, press, pulse/recovery with various lags). All models that include an EVOS impact also include density dependence (the sockeye models with EVOS allowed density dependence to vary by population). For each species, the best model and all models within 1 log-likelihood unit are highlighted in bold (the best model only being defined for this particular table—all results are included in Table 1). Lag-1 impacts were not considered on Chinook and sockeye, as these species generally migrate to the ocean in their second year of life. (DOCX) [file pone.0172898.s007.docx]

| **Model** | **Pink** | **Chinook** | **Sockeye** | **Herring** |
| --- | --- | --- | --- | --- |
| **Null (productivity constant)** | **58.622** | 50.35 | 212.593 | 171.821 |
| **1 Ricker 'b' estimated** | **58.735** | 40.332 | 208.102 | **153.545** |
| **Ricker 'b' varies by population** | -- | -- | **197.278** | -- |
| **EVOS pulse (lag 0)** | 61.48 | 43.287 | **197.784** | 154.744 |
| **EVOS press (lag 0)** | 60.246 | 31.46 | 199.514 | 156.402 |
| **EVOS pulse/recovery (lag 0)** | 59.827 | **29.643** | 199.876 | 156.201 |
| **EVOS pulse (lag 1)** | 59.602 | -- | -- | 154.587 |
| **EVOS press (lag 1)** | 61.674 | -- | -- | 155.622 |
| **EVOS pulse/recovery (lag 1)** | 61.489 | -- | -- | 155.052 |
| **EVOS pulse (lag 2)** | 61.522 | 40.52 | 199.632 | 154.826 |
| **EVOS press (lag 2)** | 61.415 | 37.569 | 199.977 | **153.177** |
| **EVOS pulse/recovery (lag 2)** | 61.168 | 37.375 | 199.914 | **152.433** |
