# Supplementary material for: Evaluating signals of oil spill impacts, climate, and species interactions in Pacific herring and Pacific salmon populations in Prince William Sound and Copper River, Alaska
Source: PLoS One. 2017 Mar 15;12(3):e0172898. doi: 10.1371/journal.pone.0172898 (PMC5351843; doi:10.1371/journal.pone.0172898)
Supplement: S4 Table — Table of model selection values (AICc) comparing models without covariates (i.e. models presented in S1 Table) to models that also estimate an impact of juvenile competition. All models with juvenile competition included also include density dependence (the sockeye models with juvenile competition allowed density dependence to vary by population). For each species, the best model and all models within 1 log-likelihood unit are highlighted in bold (the best model only being defined for this particular table—all results are included in Table 1). (DOCX) [file pone.0172898.s009.docx]

| **Model** | **Pink** | **Chinook** | **Sockeye** | **Herring** |
| --- | --- | --- | --- | --- |
| **Null (productivity constant)** | **58.622** | 50.35 | 212.593 | 171.821 |
| **1 Ricker 'b' estimated** | **58.735** | 40.332 | 208.102 | **153.545** |
| **Ricker 'b' varies by population** | -- | -- | **197.278** | -- |
| **Hatchery pink releases** | **58.926** | **37.954** | 199.837 | 155.071 |
| **Hatchery chum releases** | 61.386 | 40.838 | 199.736 | 156.349 |
