# Supplementary material for: Correction: Evaluating signals of oil spill impacts, climate, and species interactions in Pacific herring and Pacific salmon populations in Prince William Sound and Copper River, Alaska
Source: PLoS One. 2018 May 22;13(5):e0197873. doi: 10.1371/journal.pone.0197873 (PMC5963782; doi:10.1371/journal.pone.0197873)
Supplement: S1 Table — Table of model selection values (AICc) comparing null models (constant productivity, or log(R/S) independent of spawners) to models that estimated density dependence via the Ricker stock-recruitment relationship. For each species, the best model and all models within 1 log-likelihood unit are highlighted in bold (the best model only being defined for this particular table—all results are included in Table 1). (DOCX) [file pone.0197873.s001.docx]

| **Model** | **Pink** | **Chinook** | **Sockeye** | **Herring** |
| --- | --- | --- | --- | --- |
| **Null (productivity constant)** | **58.622** | 50.35 | 212.593 | **171.821** |
| **1 Ricker 'b' estimated** | **58.735** | **40.332** | 208.102 | 173.359 |
| **Ricker 'b' varies by population** | -- | -- | **197.278** | -- |
