# Supplementary material for: Correction: Evaluating signals of oil spill impacts, climate, and species interactions in Pacific herring and Pacific salmon populations in Prince William Sound and Copper River, Alaska
Source: PLoS One. 2018 May 22;13(5):e0197873. doi: 10.1371/journal.pone.0197873 (PMC5963782; doi:10.1371/journal.pone.0197873)
Supplement: S3 Table — Table of model selection values (AICc) comparing models without covariates (i.e. models presented in S1 Table) to models that also estimate an impact of environmental effects. All models that include environmental predictors also include density dependence (the sockeye models with environmental effects allowed density dependence to vary by population). For each species, the best model and all models within 1 log-likelihood unit are highlighted in bold (the best model only being defined for this particular table—all results are included in Table 1). Additional details included online, https://github.com/NCEAS/pfx-covariation-pws. (DOCX) [file pone.0197873.s003.docx]

| **Model** | **Pink** | **Chinook** | **Sockeye** | **Herring** |
| --- | --- | --- | --- | --- |
| **Null (productivity constant)** | **58.622** | 50.35 | 212.593 | -- |
| **1 Ricker 'b' estimated** | **58.735** | 40.332 | 208.102 | -- |
| **Ricker 'b' varies by population** | -- | -- | **197.278** | -- |
| **SST (lag 0)** | 61.448 | 41.878 | -- | 174.509 |
| **SST (lag 1)** | 59.045 | 43.553 | -- | 174.417 |
| **SST (lag 2)** | -- | -- | 199.572 | -- |
| **Upwelling winter (lag 1)** | 61.726 | 41.112 | **199.715** | -- |
| **Upwelling winter (lag 2)** | 61.707 | 43.068 | 199.899 | -- |
| **Upwelling spring (lag 1)** | 61.71 | -- | -- | -- |
| **Upwelling spring (lag 2)** | 61.286 | -- | -- | -- |
| **Upwelling summer (lag 1)** | -- | **38.53** | -- | 175.929 |
| **Upwelling summer (lag 2)** | -- | 42.958 | -- | 175.039 |
| **Freshwater discharge (lag 0)** | 60.968 | 42.97 | 199.279 | **171.501** |
| **Freshwater discharge (lag 1)** | 61.081 | 42.048 | 200.132 | 175.977 |
