# Supplementary material for: Correction: Evaluating signals of oil spill impacts, climate, and species interactions in Pacific herring and Pacific salmon populations in Prince William Sound and Copper River, Alaska
Source: PLoS One. 2018 May 22;13(5):e0197873. doi: 10.1371/journal.pone.0197873 (PMC5963782; doi:10.1371/journal.pone.0197873)
Supplement: S5 Table — Table of model selection values (AICc) comparing models without covariates (i.e. models presented in S1 Table) to models that also estimate an impact of predation or adult competition on wild salmon productivity. All models with predation or adult competition included also include density dependence (the sockeye models with predation or adult competition allowed density dependence to vary by population). For each species, the best model and all models within 1 log-likelihood unit are highlighted in bold (the best model only being defined for this particular table—all results are included in Table 1). All salmon models used the estimated total run size of adult salmon. (DOCX) [file pone.0197873.s005.docx]

| **Model** | **Pink** | **Chinook** | **Sockeye** | **Herring** |
| --- | --- | --- | --- | --- |
| **Null (productivity constant)** | **58.622** | 50.35 | 212.593 | **171.821** |
| **1 Ricker 'b' estimated** | **58.735** | 40.332 | 208.102 | 173.359 |
| **Ricker 'b' varies by population** | -- | -- | 197.278 | -- |
| **Wild chum** | 61.693 | 42.421 | 199.215 | 175.758 |
| **Wild pink** | 61.597 | 39.51 | 198.569 | **171.129** |
| **Hatchery chum** | 61.717 | **36.107** | 199.627 | 175.301 |
| **Hatchery pink** | 60.11 | 42.034 | **186.697** | 175.844 |
| **Total pink run** | 60.728 | 43.483 | 190.197 | 174.235 |
| **Humpback whales** | -- | -- | -- | 175.078 |
